# Dopaminergic circuits underlying associative aversive learning

**DOI:** 10.3389/fnbeh.2022.1041929

**Published:** 2022-11-10

**Authors:** Daphne Zafiri, Sevil Duvarci

**Affiliations:** Institute of Neurophysiology, Neuroscience Center, Goethe University, Frankfurt, Germany

**Keywords:** aversion, fear conditioning, dopamine, amygdala, medial prefrontal cortex (mPFC), striatum, nucleus accumbens (NAc)

## Abstract

Associative aversive learning enables animals to predict and avoid threats and thus is critical for survival and adaptive behavior. Anxiety disorders are characterized with deficits in normal aversive learning mechanisms and hence understanding the neural circuits underlying aversive learning and memory has high clinical relevance. Recent studies have revealed the dopamine system as one of the key modulators of aversive learning. In this review, we highlight recent advances that provide insights into how distinct dopaminergic circuits contribute to aversive learning and memory.

## Introduction

The ability to learn that certain stimuli and situations are associated with aversive outcomes helps animals to predict and avoid danger and hence is crucial for survival. In the laboratory, associative aversive learning is most commonly studied in rodents as well as humans using Pavlovian fear conditioning (reviewed in Duvarci and Pare, 2014). In this form of learning, an initially neutral stimulus (conditioned stimulus, CS) typically a tone is paired in time with an aversive unconditioned stimulus (US), such as a mild electrical foot shock. As the CS-US association is formed, the CS acquires the ability to elicit fear responses that are associated with the US (such as behavioral freezing) so that it can elicit conditioned fear responses when later presented alone. Much evidence indicates that anxiety disorders, such as post-traumatic stress disorder (PTSD), panic disorder and phobias, result from dysregulation of normal fear learning mechanisms (Lüthi and Lüscher, 2014; Duits et al., 2015; Craske et al., 2017), and thus understanding neural mechanisms underlying aversive learning has high clinical relevance. A considerable body of research has revealed that aversive learning and memory are mediated by a distributed network of brain structures including mainly the amygdala, medial prefrontal cortex (mPFC) and hippocampus (Duvarci and Pare, 2014; Herry and Johansen, 2014; Tovote et al., 2015; Ressler and Maren, 2019).

Recent lines of research further revealed the dopamine (DA) system as a critical regulator of aversive learning and memory. DA is a neuromodulator vitally involved in a wide range of functions including motor behavior, motivation, reward learning, cognition and aversion. DA neurons are mainly located in the ventral tegmental area (VTA) and substantia nigra (SN), but are also found in the hypothalamus, periaqueductal gray/dorsal raphe (PAG/DR) as well as the retrorubral field. DAergic transmission is mediated by metabotropic DA receptors that can be classified into two main types with the DA D1-type receptors (Gs-coupled) comprised of D1 and D5 and the DA D2-type receptors (Gi-coupled) comprised of D2, D3, and D4 subtypes (Missale et al., 1998). Although the crucial role DA neurons play in reward learning is well-established (Schultz, 2016), the role DA plays in aversive learning has more recently begun to be elucidated. Importantly, DA deficient mice exhibit impaired fear conditioning indicating the critical role DA plays in aversive learning, and enhancing DAergic transmission in these mice by systemic application of the DA precursor L-DOPA or viral-mediated restoration of DA synthesis restores fear learning (Fadok et al., 2009). Pharmacological antagonism and receptor knockout (KO) studies further demonstrate that both D1- and D2-type receptors are necessary for aversive learning (Nader and LeDoux, 1999; Greba and Kokkinidis, 2000; Inoue et al., 2000; Fadok et al., 2009). Moreover, disruption of phasic firing in DA neurons by inactivating NMDA receptors selectively in DA neurons resulted in impaired aversive learning (Zweifel et al., 2009, 2011). In line with these results, some midbrain DA neurons exhibit phasic activation in response to aversive stimuli as well as to cues predicting such stimuli (Guarraci and Kapp, 1999; Brischoux et al., 2009; Matsumoto and Hikosaka, 2009; Mileykovskiy and Morales, 2011; Zweifel et al., 2011; Jo et al., 2018; Salinas-Hernández et al., 2018). Together, these findings highlight the role of DA as a crucial neuromodulator for aversive learning.

It is important to note here that the midbrain DA system is composed of functionally distinct and mostly non-overlapping subpopulations of DA neurons each of which projects mainly to a single brain region (Lammel et al., 2008; Roeper, 2013; Beier et al., 2015; Lerner et al., 2015; Menegas et al., 2015). In particular, DA neurons projecting to brain structures that constitute the brain’s fear circuitry, such as the amygdala and the mPFC, have been implicated in aversion and aversive learning. In this short review, we highlight recent findings that have revealed the DAergic circuits underlying associative aversive learning. We mainly focus our discussion on cued fear conditioning in rodents where considerable progress has been made. Recent studies have also revealed DA as a critical regulator of fear extinction. However, in this review, we will not discuss fear extinction but rather refer the reader to prior reviews (Abraham et al., 2014; Salinas-Hernández and Duvarci, 2021). We begin by discussing how DA exerts its effects on the different components of the amygdala circuitry to regulate aversive learning and memory. We next focus on the role of DA input to the mPFC in mediating aversion and aversive learning. Finally, we discuss recent studies demonstrating how DA projections to different subregions of the striatum control aversive processes as well as the potential role DA plays in striatal circuits in mediating aversive learning.

## Dopaminergic control of amygdala circuitry underlying aversive learning and memory

**FIGURE 1:**
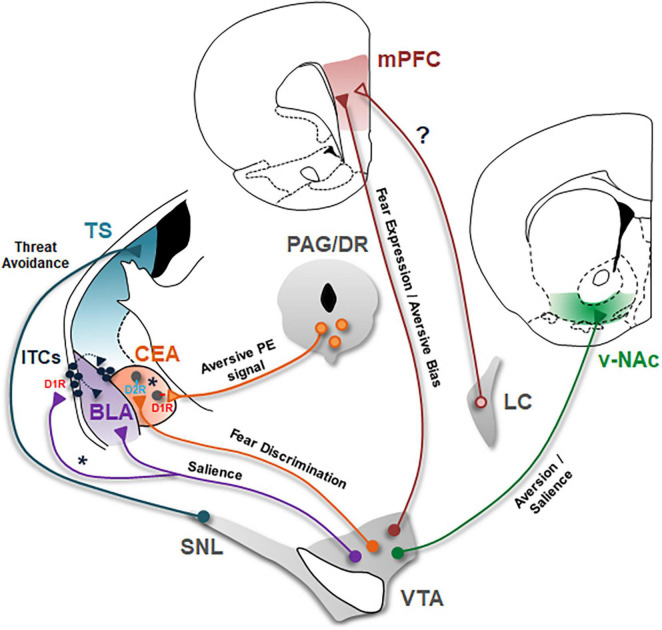
Dopaminergic circuits underlying aversive learning and memory. Schematic of the seven major DAergic projections involved in aversive learning are shown. BLA, basolateral amygdala; CEA, central nucleus of the amygdala; D1R, DA D1 receptors; D2R, DA D2 receptors; ITCs, intercalated cells; LC, locus coeruleus; mPFC, medial prefrontal cortex; PAG/DR, periaqueductal gray/dorsal raphe; SNL, substantia nigra lateralis; TS, tail of the striatum; v-NAc, ventral nucleus accumbens; VTA, ventral tegmental area. VTA DA projection to BLA encodes salience of stimuli during associative learning. Asterisk (*) indicate a possible DAergic projection to lateral ITCs in mediating disinhibition of BLA and dorsomedial ITCs during aversive learning. The role of this projection in aversive learning remains to be tested. CEA receives DA input from both VTA and PAG/DR. Whereas, PAG/DR DA projections encode an aversive prediction error signal to drive aversive learning, VTA DA projections mediate fear discrimination. Asterisk (*) indicate a possible scenario in which PAG/DR and VTA DA neurons selectively innervate D1R- and D2R- expressing CEA neurons, respectively. Whether different subtypes of CEA neurons indeed receive separate inputs from these two different DA projections is an open question that remains to be investigated. DA input to mPFC originates from both VTA and LC. VTA DA projections are critical for expression of conditioned fear responses. In addition, this DA input to mPFC biases behavior toward aversion. On the other hand, the role of DA input from LC neurons in aversive learning needs to be investigated (?). VTA DA projections to v-NAc are implicated in aversion and likely encode motivational salience. Whether this DA projection is crucial for associative aversive learning remains to be tested. SNL DA neurons projecting to TS mediate threat avoidance. It will be important to investigate whether this DA projection is critically involved in associative aversive learning.

The amygdala is a key structure underlying aversive learning and memory formation. Particularly, within the amygdala circuitry, the basolateral amygdala (BLA), consisting of the lateral and basal nuclei, the central nucleus of the amygdala (CEA) and the intercalated cells of the amygdala (ITCs) are the critical nuclei required for acquisition, consolidation and expression of aversive memories (Figure 1; Johansen et al., 2011; Duvarci and Pare, 2014; Herry and Johansen, 2014; Tovote et al., 2015; Ressler and Maren, 2019). Both D1- and D2-type DA receptors are expressed abundantly in the amygdala (Meador-Woodruff et al., 1991) and early pharmacological studies have established that both DA receptor types in the amygdala are critically involved in aversive learning (Pezze and Feldon, 2004).

### Basolateral amygdala

Studies administering D1 and D2 receptor agonists and antagonists in the BLA indicated the essential role these receptors play in BLA for aversive learning and memory (Lamont and Kokkinidis, 1998; Guarraci et al., 1999; Nader and LeDoux, 1999; Greba and Kokkinidis, 2000; Greba et al., 2001). Plasticity in the synapses carrying sensory information about the CS and the US underlies acquisition and consolidation of conditioned fear memories in BLA (Sigurdsson et al., 2007; Ressler and Maren, 2019). As a result, BLA neurons exhibit increased CS-evoked responses following fear conditioning (Quirk et al., 1995; Rogan et al., 1997; Collins and Pare, 2000; Repa et al., 2001; Goosens et al., 2003; Herry et al., 2008; Amano et al., 2011). Importantly, activation of DA receptors is critical for development of CS-evoked responses in BLA neurons (Rosenkranz and Grace, 2002). Consistent with this, DA has been shown to increase excitability of principal BLA neurons (Kröner et al., 2005; Yamamoto et al., 2007; Li et al., 2011). Other potential targets of DA in the BLA microcircuitry are GABAergic interneurons. Inhibitory circuits have been shown to play an essential role in gating aversive learning in BLA (Krabbe et al., 2018). In line with this, DA suppresses feedforward inhibition onto principal BLA neurons through D2-receptor mediated inhibition of fast-spiking interneurons, and as a result facilitates synaptic plasticity in the BLA (Bissière et al., 2003). Yet, there are several distinct subtypes of interneurons in the BLA that are critically involved in aversive learning (Krabbe et al., 2018). How DA regulates the activity of each of these interneuron subtypes is largely unknown and will be an important question for future research.

Recent findings indicate that the source of DA mediating aversive learning in BLA originates from DA neurons located in the VTA (Figure 1; Lutas et al., 2019; Tang et al., 2020). These studies demonstrated that VTA DA neurons projecting to BLA are activated by aversive USs as well as CSs associated with them (Lutas et al., 2019; Tang et al., 2020); furthermore, optogenetic inhibition of VTA DA projections to BLA impairs aversive learning (Tang et al., 2020). This raises the question of whether these BLA projecting VTA DA neurons are activated exclusively by aversive events and thus mediate only aversive learning. The BLA is also important for reward learning which is mediated by a subpopulation of neurons that is distinct from those mediating aversive learning, based on gene expression pattern and projection targets (Namburi et al., 2015; Kim J. et al., 2016). Intriguingly, individual VTA DA axons in the BLA are activated by both reward and aversive events as well as CSs predicting them (Lutas et al., 2019), suggesting that activation of the same DA neuron mediates both aversive and reward learning. These results suggest that BLA-projecting VTA DA neurons respond to the salience of stimuli, rather than their positive or negative value; and mediate associative learning in general for both negative and positive outcomes.

### Central nucleus of the amygdala

As mentioned earlier, the second crucial component of the amygdala circuitry underlying aversive learning and memory is the CEA. Much evidence indicates that plastic changes in the activity of CEA neurons mediate acquisition, consolidation, and expression of conditioned fear responses (Wilensky et al., 2006; Zimmerman et al., 2007; Ciocchi et al., 2010; Haubensak et al., 2010; Duvarci et al., 2011; Li et al., 2011; Fadok et al., 2018). In this review, we will not discuss the CEA microcircuitry and the different subtypes of CEA neurons but rather refer the reader to prior reviews (Duvarci and Pare, 2014; Fadok et al., 2018; Kong and Zweifel, 2021). Like BLA, CEA receives DA inputs originating from the VTA but also from the PAG/DR (Figure 1; Hasue and Shammah-Lagnado, 2002). Interestingly, recent studies investigating the function of these DA projections to CEA have revealed that these two distinct DA inputs play different roles in aversive learning and memory. Notably, PAG/DR DA neurons projecting to CEA encode a positive prediction error (PE) signal for aversive stimuli that drives associative aversive learning in CEA (Groessl et al., 2018). On the other hand, optogenetic inhibition of VTA DA projections to CEA does not have an effect on acquisition of conditioned fear suggesting that this DA projection is not necessary for associative aversive learning (Jo et al., 2018; Tang et al., 2020). VTA DA projections to CEA are instead critical for fear discrimination—that is discriminating cues associated with danger from cues associated with safety (Jo et al., 2018). Together, these findings indicate that these two distinct DA projections to CEA have different roles in aversive learning.

These observations raise the question of whether different subtypes of CEA neurons receive separate inputs from these different DA projections. Anatomical studies demonstrate that although D2 receptors are more abundant, both D1 and D2 receptors are expressed in CEA (Weiner et al., 1991; Perez de la Mora et al., 2012; McCullough et al., 2018). Moreover, expression of D1 and D2 receptors for the most part does not overlap in CEA neurons (McCullough et al., 2018). Interestingly, a recent study demonstrated that activation of D2 receptors in the CEA mediates fear discrimination (De Bundel et al., 2016). It would therefore be interesting to investigate whether VTA DA neurons innervate preferentially the D2 receptor-expressing neurons in CEA. On the other hand, PAG/DR DA neurons exhibit phasic activation to aversive USs as well as CSs associated with an aversive US (Groessl et al., 2018) suggesting that this DA input would activate preferentially the D1 receptor expressing CEA neurons during aversive learning. Tracing of monosynaptic inputs to genetically defined CEA neurons will be necessary to determine whether they receive differential inputs from VTA and PAG/DR DA neurons.

### Intercalated cells of the amygdala

Another important target of DA in the amygdala circuitry are the ITCs which are a network of interconnected GABAergic cell groups located in the external and the intermediate capsules surrounding the BLA (Figure 1). The source of DA input to ITCs has largely remained elusive due to lack of molecular tools that can selectively target these neurons. However, recent studies suggest that DA input to ITCs originates from VTA/SN, similar to BLA (Ferrazzo et al., 2019; Aksoy-Aksel et al., 2021). D1, but not D2, receptors are abundantly expressed in the ITCs (Jacobsen et al., 2006). D1 receptors are typically Gs-coupled receptors and when activated they are expected to function in an excitatory fashion (Missale et al., 1998). Interestingly, D1 receptor signaling in ITCs is unusual in that activation of D1 receptors hyperpolarizes ITCs and inhibits these neurons (Marowsky et al., 2005; Mańko et al., 2011). ITCs are ideally positioned to gate sensory inputs to BLA as they exert feedforward and also feedback inhibition onto principal BLA neurons (Figure 1; Marowsky et al., 2005; Asede et al., 2015, 2021). DA inhibits these neurons and hence can facilitate synaptic plasticity in BLA (Marowsky et al., 2005). Furthermore, medial ITCs, located in the intermediate capsule, also gate the information flow from BLA to CEA. They receive excitatory input from BLA and send inhibitory projections to CEA, and thus mediate feedforward inhibition of CEA (Royer et al., 1999; Paré et al., 2003; Mańko et al., 2011; Gregoriou et al., 2019). In particular, plasticity in the dorsally located medial ITCs (dm-ITCs) has been implicated in aversive learning (Busti et al., 2011; Asede et al., 2015; Kwon et al., 2015). dm-ITCs are activated during acquisition and retrieval of conditioned fear (Busti et al., 2011; Hagihara et al., 2021). Both aversive USs and CSs predicting an aversive US excite dm-ITCs (Hagihara et al., 2021). Bidirectional chemogenetic manipulations during fear retrieval further demonstrated that activation of dm-ITCs is critical for expression of conditioned fear (Hagihara et al., 2021). Since DA inhibits ITCs, it is unlikely that dm-ITCs receive direct DA input during fear acquisition and expression. However, different ITC clusters are connected and exert feedforward inhibition onto each other (Royer et al., 1999, 2000; Paré et al., 2003; Asede et al., 2015, 2021). Notably, a recent study demonstrated that lateral ITCs (l-ITCs) located in the external capsule send feedforward inhibition onto dm-ITCs and DA application reduces this inhibition onto dm-ITCs (Aksoy-Aksel et al., 2021). These findings suggest that DA likely inhibits l-ITCs, dampening the feedforward inhibition these neurons exert on BLA and dm-ITCs, and thus indirectly activates BLA and dm-ITC neurons during aversive learning (Figure 1). Based on this scenario, DA neurons would be expected to differentially innervate and modulate distinct clusters of ITCs. How DA input regulates activity of each ITC cluster to mediate aversive learning is therefore an important question for future studies.

## Role of dopamine in the medial prefrontal cortex in mediating aversive learning

Considerable research has indicated that mPFC is a brain region critically involved in aversive learning. In particular, the prelimbic subregion of mPFC mediates expression of conditioned fear responses (Sotres-Bayon and Quirk, 2010; Giustino and Maren, 2015; Tovote et al., 2015; Rozeske and Herry, 2018). Early studies investigating the effect of aversive events and stressors in mPFC revealed that such events enhance prefrontal DA levels (Abercrombie et al., 1989; Sorg and Kalivas, 1993; Sullivan and Gratton, 1998). Importantly, an increase in DA release has also been observed in mPFC during fear conditioning (Wilkinson et al., 1998; Feenstra, 2000; Feenstra et al., 2001). Consistent with this, pharmacological studies demonstrated that activation of DA receptors in mPFC is required for expression of conditioned fear (Pezze et al., 2003; Jing Li et al., 2018). Together, these findings demonstrate the crucial role DA plays in aversive learning and memory in mPFC.

Recent studies reveal that DA input to mPFC originates from both VTA DA neurons and noradrenaline (NA) neurons located in the locus coeruleus (LC; Figure 1; Lammel et al., 2008; Beier et al., 2015; Devoto et al., 2020). Notably, mPFC-projecting VTA DA neurons have been implicated in aversive processing (Lammel et al., 2011, 2012; Gunaydin et al., 2014). Fiber photometry recordings of VTA DA terminals in mPFC demonstrate that these DA cells are selectively activated by aversive events, but not rewards (Kim C. K. et al., 2016). Consistent with this, a recent study revealed that DA increases the signal-to-noise ratio of responses in particular to aversive stimuli in mPFC. This study further showed that optogenetic activation of VTA DA terminals in mPFC biased behavioral responses to aversive stimuli in an associative stimulus competition task where aversive and reward CSs were presented simultaneously (Vander Weele et al., 2018). Together, these findings suggest that DA released from VTA DA neurons is a critical modulator of aversive processing and acts as a pro-aversive signal in the mPFC. On the other hand, the role that DA input from LC NA neurons plays during aversive learning has remained elusive (Figure 1). Whereas VTA DA neurons target the deep layers, LC NA neurons innervate the superficial layers of mPFC (Vander Weele et al., 2018). This differential innervation pattern suggests that DA released from LC NA neurons might mediate functions different from DA released from VTA. It will be important for future studies to examine the different roles these two distinct DA inputs play in mPFC during aversive learning.

## Dopaminergic projections to the striatum underlying aversive processing

Recent studies using the latest cell type- and projection-specific techniques have highlighted two regions of the striatum, the nucleus accumbens (NAc) and the posterior tail of the striatum (TS), as the striatal targets of aversion encoding DA neurons. As mentioned earlier, it is well-established that DA neurons encode reward PE signals to drive reward learning and in particular DA projections to NAc constitute the brain’s canonical reward circuitry (Wise, 2002; Schultz, 2016). More recent studies, however, have revealed that DA projections to NAc are not homogeneous, in that different subpopulations of DA neurons indeed project to distinct subregions of NAc and mediate different functions (Roeper, 2013). Earlier studies examining DA release suggested an involvement of DA in NAc during aversive processing (Young, 2004; Badrinarayan et al., 2012; Budygin et al., 2012; Oleson et al., 2012; Carelli and West, 2014). Two recent studies performing fiber photometry recordings of calcium transients in DA neuron terminals across different subregions of the NAc have revealed that in particular the ventral NAc (vNAc) projecting DA neurons mediate aversive processes (Figure 1; de Jong et al., 2019; Yuan et al., 2019). These two studies demonstrated that DA projections to vNAc are activated by aversive stimuli and CSs that predict them. However, similar to BLA projecting DA neurons, these DA terminals exhibit increased responses also to rewards (de Jong et al., 2019; Yuan et al., 2019) suggesting that they likely encode motivational salience rather than aversiveness *per se*. However, since recordings of bulk calcium transients from DA terminals in vNAc were performed in these studies, it is unclear whether the same DA neurons are activated by both rewards and aversive events. Studies recording the activity of vNAc-projecting DA neurons at single cell resolution will be necessary to address this question. Importantly, although vNAc-projecting DA neurons exhibit increased CS-evoked responses following pairing of the CS with an aversive US (de Jong et al., 2019), whether this activity is indeed required for acquisition and expression of associative aversive memories is not known. Causal manipulations investigating the necessity of DA projections to vNAc during aversive learning will be crucial to address this question.

The second striatal region that has lately been implicated in aversive processing is the posterior tail of the dorsal striatum (TS). Earlier studies investigating the role of dorsal striatum (DS) and DA projections to DS have particularly implicated dorsolateral striatum (DLS) in aversive learning. Notably, DLS-projecting DA neurons have been shown to be activated by aversive stimuli (Lerner et al., 2015). However, the causal contribution of DS-projecting DA neurons in aversive learning has remained elusive. For more detailed information on the role of DS in aversion, the reader is referred to prior reviews (Stanley et al., 2021). In this review, we will concentrate on DA projections to TS (Figure 1) where recent progress has been made. Indeed, recent studies have identified a unique subpopulation of DA neurons that project to the TS, located in the most lateral part of SN (SNL) and which exhibit a distinct input-output organization (Menegas et al., 2015). These DA neurons respond particularly strongly to novel and aversive stimuli, but only weakly to rewards. Consistent with this, they encode an aversive PE rather than a reward PE signal and exhibit CS-evoked responses following pairing of the CS with an aversive US (Menegas et al., 2017, 2018). Selective ablation or activation of these DA neurons further demonstrates that they are involved in threat avoidance (Menegas et al., 2018). However, whether this DA projection is crucial for aversive learning is currently unknown. Considering these DA neurons have been demonstrated to encode an aversive PE signal (Menegas et al., 2017, 2018), it will be important to investigate whether they are critically involved in associative aversive learning.

## Concluding remarks

Recent advances in cell type- and projection-specific strategies in recording and manipulating neuronal activity have led to considerable progress in understanding the role of defined DAergic circuits in aversive learning. The studies reviewed above provide insights into how DA neurons mediate different aspects of aversive processing through their projections to the amygdala, mPFC and the striatum. The main conclusions of these studies and outstanding questions are summarized in Figure 1. In the amygdala, DA projections to BLA encode salience of stimuli to mediate associative learning. However, how DA regulates the activity of BLA neurons, in particular the activity of different subtypes of inhibitory interneurons in mediating aversive learning is incompletely understood. Likewise, how DA input controls the activity of different ITC clusters during aversive learning has also remained elusive. An important outstanding question for future research is whether DA neurons differentially innervate distinct clusters of ITCs to mediate aversive learning. CEA receives two distinct DA inputs from VTA and PAG/DR which play different roles in aversive learning. Whereas projections from PAG/DR encode an aversive PE signal to drive associative aversive learning, projections from VTA mediate fear discrimination. Whether different subtypes of CEA neurons, based on their DA receptor expression, receive separate inputs from these two distinct DA projections is an open question remains to be investigated. In the mPFC, DA input from VTA is critical for expression of conditioned fear responses and furthermore biases behavior toward aversion. mPFC receives DA input also from LC but the role of this input during aversive learning is largely unknown. In the striatum, two striatal subregions have recently emerged as the targets of DA neurons mediating aversive processing. Whereas DA projections to vNAc are involved in aversion and likely encode motivational salience, DA projections to TS mediate threat avoidance. Yet, whether these DA projections to the striatum are crucial for associative aversive learning remains to be an important outstanding question for future studies. Overall, the findings summarized in this review highlight DA as a critical modulator of aversive learning. Understanding the key role DA plays in aversive learning has high clinical significance since dysregulation of aversive learning mechanisms is a hallmark of human anxiety disorders. In particular, elucidating how DA mediates different aspects of aversive processing through its actions in distinct brain circuits can help develop novel therapeutic strategies in the treatment of anxiety disorders.

## Author contributions

DZ and SD wrote the manuscript. Both authors contributed to the article and approved the submitted version.
